# Electrocardiography on admission is associated with poor outcomes in coronavirus disease 2019 (COVID‐19) patients: A systematic review and meta‐analysis

**DOI:** 10.1002/joa3.12573

**Published:** 2021-06-14

**Authors:** Mochamad Yusuf Alsagaff, Yudi Her Oktaviono, Budi Baktijasa Dharmadjati, Achmad Lefi, Makhyan Jibril Al‐Farabi, Parama Gandi, Bagas Adhimurda Marsudi, Yusuf Azmi

**Affiliations:** ^1^ Department of Cardiology and Vascular Medicine Faculty of Medicine Soetomo General Hospital Universitas Airlangga Surabaya Indonesia; ^2^ Department of Cardiology and Vascular Medicine Faculty of Medicine Harapan Kita National Heart Center Universitas Indonesia Jakarta Indonesia; ^3^ Faculty of Medicine Universitas Airlangga Surabaya Indonesia

**Keywords:** COVID‐19, electrocardiogram, ICU admission, mortality, severe illness

## Abstract

**Background:**

Electrocardiogram (ECG) is a widely accessible diagnostic tool that can easily be obtained on admission and can reduce excessive contact with coronavirus disease 2019 (COVID‐19) patients. A systematic review and meta‐analysis were performed to evaluate the latest evidence on the association of ECG on admission and the poor outcomes in COVID‐19.

**Methods:**

A literature search was conducted on online databases for observational studies evaluating ECG parameters and composite poor outcomes comprising ICU admission, severe illness, and mortality in COVID‐19 patients.

**Results:**

A total of 2,539 patients from seven studies were included in this analysis. Pooled analysis showed that a longer corrected QT (QTc) interval and more frequent prolonged QTc interval were associated with composite poor outcome ([WMD 6.04 [2.62‐9.45], *P* = .001; *I* ^2^:0%] and [RR 1.89 [1.52‐2.36], *P* < .001; *I* ^2^:17%], respectively). Patients with poor outcome had a longer QRS duration and a faster heart rate compared with patients with good outcome ([WMD 2.03 [0.20‐3.87], *P* = .030; *I* ^2^:46.1%] and [WMD 5.96 [0.96‐10.95], *P* = .019; *I* ^2^:55.9%], respectively). The incidence of left bundle branch block (LBBB), premature atrial contraction (PAC), and premature ventricular contraction (PVC) were higher in patients with poor outcome ([RR 2.55 [1.19‐5.47], *P* = .016; *I* ^2^:65.9%]; [RR 1.94 [1.32‐2.86], *P* = .001; *I* ^2^:62.8%]; and [RR 1.84 [1.075‐3.17], *P* = .026; *I* ^2^:70.6%], respectively). T‐wave inversion and ST‐depression were more frequent in patients with poor outcome ([RR 1.68 [1.31‐2.15], *P* < .001; *I* ^2^:14.3%] and [RR 1.61 [1.31‐2.00], *P* < .001; *I* ^2^:49.5%], respectively).

**Conclusion:**

Most ECG abnormalities on admission are significantly associated with an increased composite poor outcome in patients with COVID‐19.

## Graphical abstract

Electrocardiography abnormalities on admission, including longer QTc interval and prolonged QTc interval, longer QRS duration, a faster heart rate, the presence of LBBB, PAC, PVC, T‐wave inversion, and ST‐depression are significantly associated with an increased composite poor outcome in patients with COVID‐19.

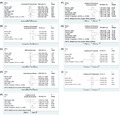

## 1 INTRODUCTION

On January 30, 2020, the World Health Organization (WHO) declared 2019 coronavirus disease (COVID‐19), an infectious disease caused by Severe Acute Respiratory Syndrome‐Coronavirus‐2 (SARS‐CoV‐2), as a pandemic.^1^ As of November 22, 2020, it was reported that more than 57.8 million people worldwide were infected with COVID‐19, causing more than 1.3 million fatalities.^2^ While most of the focus is on diseases and complications of the lung, one cannot ignore myocardial injury as it can worsen the prognosis and increase mortality.^3^, ^4^ SARS‐CoV‐2 binds to the host cell surface via the angiotensin‐converting enzyme 2 (ACE2) receptor, which causes pulmonary infection and cardiac complications of acute myocardial injury (27.8%) and arrhythmias (44.4%).^5^, ^6^, ^7^

Due to the severe complications in the heart, a diagnostic tool is needed to help predict the condition of the patients quickly during admission. Electrocardiography (ECG) is a widely available diagnostic tool that can be done immediately and can reduce excessive contact with the patient. Previous studies have reported that many COVID‐19 patients present with ECG alterations associated with cardiac involvement, such as a prolonged QTc interval, ST‐segment abnormalities, atrial and ventricular arrhythmias, and conduction block.^8^, ^9^ Therefore, we performed a systematic review and meta‐analysis to evaluate the latest evidence on the association of ECG on admission and the poor outcomes in COVID‐19.

## 2 METHODS

### 2.1 Eligibility criteria

We included all studies evaluating ECG parameters on admission and outcomes comprising ICU admission, severe illness, and mortality in patients who tested positive for SARS‐CoV‐2 using the reverse transcription‐polymerase chain reaction (RT‐PCR) test. Unpublished studies, animal or in‐vitro studies, review articles, case reports, non‐English articles, and studies with irrelevant or non‐extractable results were excluded from the analysis.

### 2.2 Search strategy and study selection

We conducted a systematic literature search for January 1, 2020, to November 1, 2020, from PubMed, the Cochrane Library Database, and Europe PMC using the search strategy shown in Table S1. After the initial search, duplicate articles were removed. The abstracts and titles of the remaining articles were screened by two authors (MJA and YA) independently. Subsequently, the relevant articles in the full text were assessed based on the eligibility criteria. Disagreements were resolved by conferring with the senior writer (MYA). This research was conducted following the Preferred Reporting Item for Systematic Reviews and Meta‐Analysis (PRISMA) statement.

### 2.3 Data collection process

Two authors (MJA and YA) conducted data extraction independently using standardized form extraction consisting of the author, date of publication, study design, number and characteristics of samples, ECG parameters, ICU admission, severe illness, and mortality. The ECG parameters included corrected QT (QTc) interval, prolonged QTc interval, QRS duration, PR interval, heart rate, right bundle branch block (RBBB), left bundle branch block (LBBB), premature atrial contraction (PAC), premature ventricular contraction (PVC), T‐wave inversion, ST‐depression, and ST‐elevation. The Bazett formula (QTc = QT/(√RR)) was used to calculate the QTc interval.^10^ The outcome of interest was composite poor outcomes, including ICU admission, severe illness, and mortality. The severity of the disease was defined in the diagnosis and treatment guidelines of adults with community‐acquired pneumonia.^11^ We used mean ± standard deviation (SD) and frequency (percentage) to present the distribution of the categorical and continuous variables, respectively.

### 2.4 Quality assessment

The risk of bias and the quality of included studies were assessed using the Newcastle‐Ottawa score (NOS)^12^ by all authors independently, and discrepancies were resolved through discussion. This scoring system consists of three domains: sample selection, comparability of cohorts, and outcomes assessment (Table S2).

### 2.5 Data analysis

Stata software V.14.0 (College Station) was used for meta‐analysis. Pooled effect estimates of the continuous and dichotomous variables were reported as weighted means differences (WMD) and relative risk (RR), respectively. We used the fixed‐effects models for pooled analysis with low heterogeneity (*I* ^2^ statistic <50% or *P*‐value >.1), while the random‐effects models were used for pooled analysis with high heterogeneity (*I* ^2^ statistic >50% or *P*‐value ≤.1). For other analyses, *P*‐value <.05 was determined as statistical significance. Subgroup analysis was performed for the parameter of the QTc interval. The publication bias was evaluated qualitatively using funnel‐plot analysis. To evaluate the small‐study effects on dichotomous and continuous variables, we used the regression‐based Harbord test and Egger test, respectively.

## 3 RESULTS

### 3.1 Study characteristics

**FIGURE 1:**
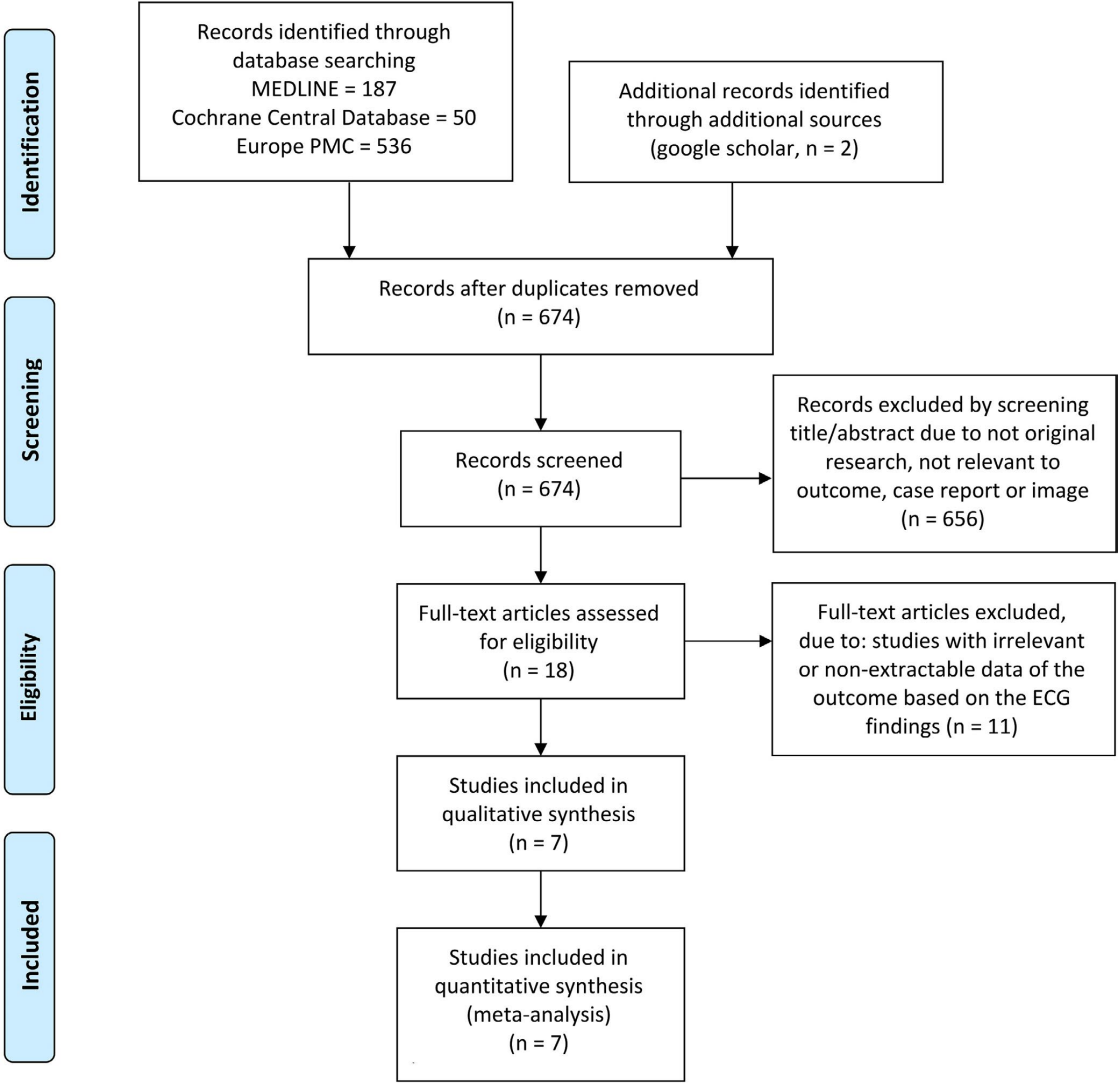
PRISMA flowchart

We identified 775 articles from the initial search, and 674 articles remained after the duplication was removed. Screening on titles and abstracts excluded 661 articles, and the remaining 18 full‐text articles were assessed according to eligibility criteria. As a result, seven studies^13^, ^14^, ^15^, ^16^, ^17^, ^18^, ^19^ with a total of 2,539 patients were subjected to qualitative analysis and meta‐analysis (Figure 1; Table 1). Quality assessment with NOS showed that included studies were of good quality (Table S1).

**TABLE 1:** Characteristics of the included studies

Authors	Study design	Samples (good/poor outcome group)	Age means (SD)	Male (%)	Time of ECG recording	ECG parameters	Outcome	NOS
Barman, 2020^13^	Observational retrospective	219 (95/124)	61.1	64%	At admission	Heart rate, PR interval, QRS duration, QTc interval, prolonged QTc (>500 ms), ST‐depression, T‐wave inversion, RBBB	Severe illness	9
Lanza, 2020^14^	Observational retrospective	44/280	77.8 (9)	66%	At admission	Heart rate, PR interval, QRS duration, QTc interval, prolonged QTc (≥460 ms in women; ≥450 ms in men), ST‐depression, T‐wave inversion, RBBB	Mortality	9
Li, 2020^15^	Observational retrospective	135 (23/112)	61.3 (18)	51%	At admission	Heart rate, PR interval, QRS duration, QTc interval, prolonged QTc (≥460 ms in women; ≥450 ms in men), PAC, PVC, RBBB	ICU admission	9
McCullough, 2020^16^	Observational retrospective	756 (666/90)	63.3 (16)	63%	At or near hospital admission	Heart rate, QTc interval, ST‐elevation, T‐wave inversion, PAC, PVC, RBBB, LBBB	Mortality	9
Moey, 2020^17^	Observational retrospective	95 (51/44)	60 (16.4)	41%	At admission and during hospitalization	PR interval, QRS duration, QTc interval	ICU admission	8
Poterucha, 2020^18^	Observational retrospective	887 (556/331)	64.1 (17)	58%	Within two days of admission or diagnosis	PR interval, QRS duration, QTc interval, prolonged QTc (≥500 ms), ST‐depression, ST‐elevation, PAC, PVC, RBBB, LBBB	Ventilator requirement, mortality	8
Rath, 2020^19^	Observational retrospective	123 (107/16)	68 (15)	63%	At admission	Heart rate, QRS duration, QTc interval, ST‐depression, ST‐elevation, T‐wave inversion, RBBB, LBBB	Mortality	8

Abbreviations: ECG, electrocardiogram; LBBB, left bundle branch block; ms, millisecond; NOS, Newcastle‐Ottawa Scale; PAC, premature atrial contraction; PVC, premature ventricular contraction; QTc, corrected QT (QTc) interval; RBBB, right bundle branch block.

### 3.2 Electrocardiogram parameters and outcome

**FIGURE 2:**
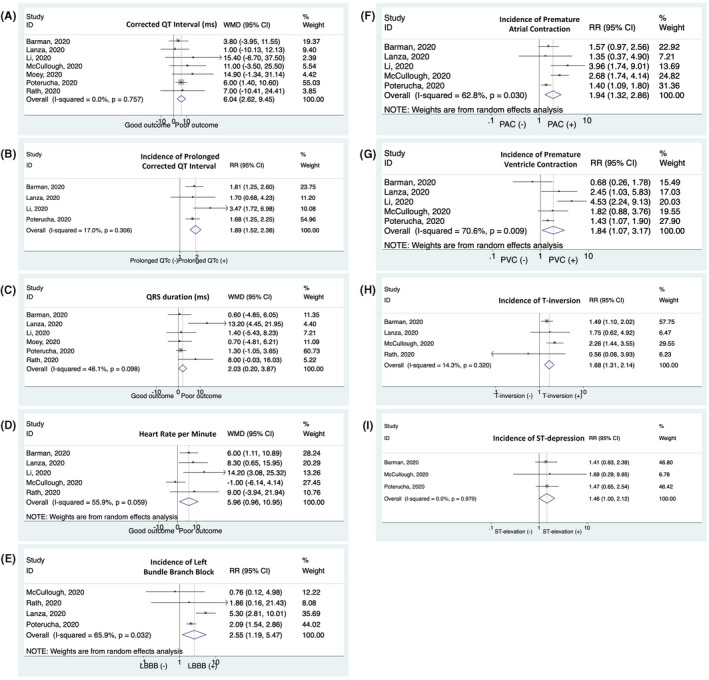
Several ECG findings and the outcome of COVID‐19. COVID‐19 patients presenting with (A) a longer corrected QT interval, (B) prolonged QTc, (C) a longer QRS duration, (D) a faster heart rate, (E) left bundle branch block, (F) premature atrial contraction, (G) premature ventricular contraction, (H) T‐wave inversion, and (I) ST‐depression have an increased risk of composite poor outcome

Meta‐analysis showed that longer QTc interval was found in patients with poor outcome (weighted means difference, WMD 6.04 [2.62‐9.45], *P* = .001; *I* ^2^:0%) compared with patients with good outcome. Prolonged QTc interval was associated with composite poor outcome (relative risks, RR 1.89 [1.52‐2.36], *P* < .001; *I* ^2^:17%). Patient with poor outcome had also longer QRS duration and faster heart rate than those with good outcome ([WMD 2.03 [0.20‐3.87], *P* = .030; *I* ^2^:46.1%] and [WMD 5.96 [0.96‐10.95], *P* = .019; *I* ^2^:55.9%], respectively). The incidence of LBBB, PAC, and PVC on admission ECG was higher in patients with poor outcome ([RR 2.55 [1.19‐5.47], *P* = .016; *I* ^2^:65.9%]; [RR 1.94 [1.32‐2.86], *P* = .001; *I* ^2^:62.8%]; and [RR 1.84 [1.075‐3.17], *P* =.026; *I* ^2^:70.6%], respectively). ST‐segment changes including T‐wave inversion and ST‐depression were also associated with composite poor outcome ([RR 1.68 [1.31‐2.15], *P* < .001; *I* ^2^:14.3%] and [RR 1.61 [1.31‐2.00], *P* < .001; *I* ^2^:49.5%], respectively; Figure 2). Other ECG parameters such as PR interval and incidence of RBBB and ST‐elevation were not significantly associated with poor outcomes (Figure S1).

### 3.3 Publication bias

**FIGURE 3:**
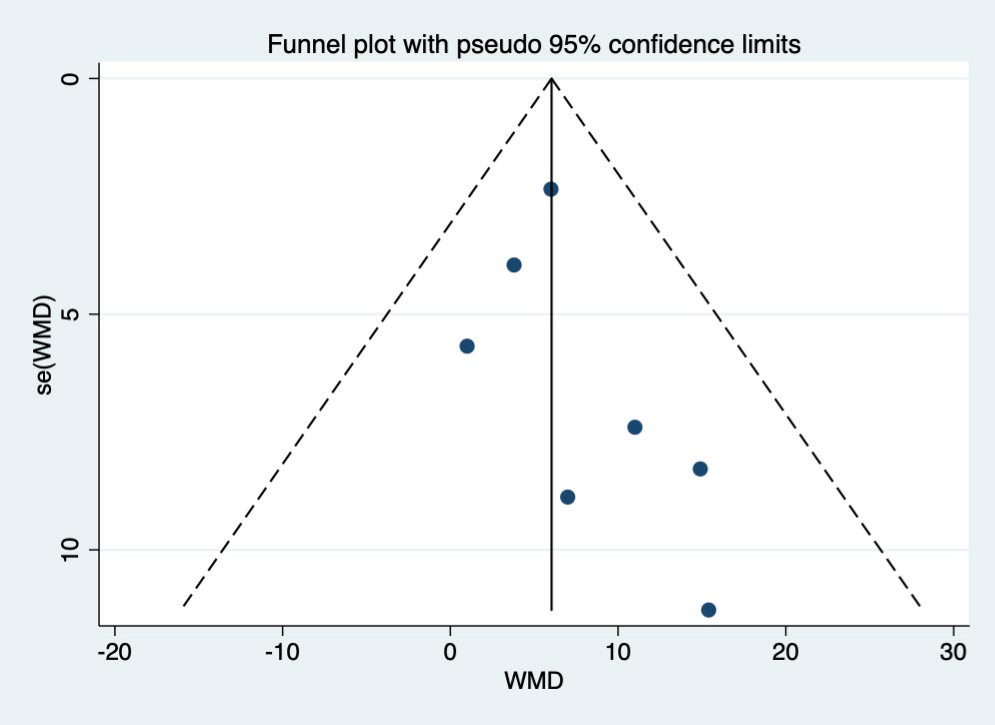
Funnel‐plot analysis. WMD, weighted mean differences

The visual assessment of the funnel plot showed an asymmetrical shape for the analysis of the QTc interval, which indicated the possibility of publication bias (Figure 3). However, quantitative analysis using regression‐based Egger's test for the same variable showed no significant result of small‐study effects (*P* = .262). Regression‐based Harbord's test for other ECG parameters and composite poor outcome also ﻿showed no significant result of small‐study effects.

## 4 DISCUSSION

Cardiac injury is one of the complications that represent severe COVID‐19,^3^ and the ECG is still the simplest tool to assess myocardial involvement. This meta‐analysis revealed that, on admission ECG, patients with poor outcomes tend to have a longer QTc interval, more frequent prolonged QTc interval, longer QRS duration, faster heart rate, higher incidence of LBBB, PAC, PVC, T‐wave inversion, and ST‐depression compared with patients with a good outcome. Several previous reviews have described the manifestations of COVID‐19 patients on ECG abnormalities and the effect of medications such as chloroquine, hydroxychloroquine, and azithromycin on QTc prolongation and its association to poor outcomes. Other studies in patients who were not treated with the drugs mentioned above have found that ECG findings associated with mortality and morbidity limited to PR interval changes, axis changes, unspecific ST‐T abnormalities, and cardiac arrhythmias such as atrial fibrillation (AF), supraventricular tachycardia (SVT), ventricular tachycardia (VT), and ventricular fibrillation (VF).^20^, ^21^ To the best of our knowledge, this is the first systematic review and meta‐analysis to describe the abnormality of each ECG parameter on admission and to evaluate its association with the outcomes of COVID‐19 patients and adds several new findings, where prolonged QRS findings, LBBB, and PACs and PVCs are associated with worse outcomes in COVID‐19 patients. Such findings should warrant caution in clinical practice as they reflect dysfunctional intracellular calcium release and eventual calcium overload resulting in after early after depolarizations (EADs) and delayed after depolarizations (DADs), which will be discussed in more depth later.

The QT interval is the ventricular period of depolarization and repolarization, depicted from the beginning of the Q wave to the end of the T wave.^22^ Abnormal prolongation of this period can cause life‐threatening ventricular arrhythmias, especially torsade de pointes (TdP).^23^ Preexisting prolonged QTc (>500 ms) is prevalent in patients with COVID‐19. In New York City hospital, prolonged QTc was found on 260 of 4250 patients (6.1%) at admission.^24^ Another study reported that nearly 10% of 623 COVID‐19 patients were admitted with a prolonged QTc interval (QTc >480 ms), and prolonged QTc was significantly associated with higher fatality rates.^25^ The present meta‐analysis showed that COVID‐19 patients with preexisting prolonged QTc tend to have poor outcomes.

Many factors contribute to a prolonged QTc interval in the patient with COVID‐19, but it is likely due to the inflammation and the over‐expression of Angiotensin 2 (AngII) as a result of SARS‐COV2 infection. In COVID‐19 patients, inflammation can be either localized to the heart in the form of myocarditis/endocarditis^26^ or spread systemically, causing a more severe systemic inflammatory response. Elevated pro‐inflammatory interleukin‐6 levels due to systemic inflammation response have a potential electrophysiological effect on ion channels that can alter the duration of action potential and the QTc interval.^27^ Additionally, SARS‐COV2 viral load and increased virus endocytosis may also play a role in the development of this finding. Endocytosis of SARS‐COV2 is mediated by Angiotensin‐converting enzyme 2 receptor (ACE2R) in the cell membrane, which is expressed abundantly on pulmonary epithelial cells, cardiomyocytes, and vascular endothelial cells.^28^ Utilization of these receptors leads to downregulation of ACE‐2R, which results in a pathway shift toward increased production of angiotensin II that binds to Angiotensin II type 1 receptor (AT1R) and Endothelin 1 receptor (ET1).^29^ These pathways result in the formation of reactive oxygen species (ROS) through the activation of Nox2 and subsequent NADPH oxidase enzyme.^30^, ^31^ Increased ROS can directly affect the heart by inducing apoptosis of several cardiac tissues, causing worsening heart failure, vascular damage, and sinus node dysfunction. Increased ROS can also directly influence CAMK‐II regulation. Pathological CAMK‐II regulation triggers the spontaneous release of electrogenic Ca^2+^ via extrusion Na^+^/Ca^2+^ exchanger, phosphorylation of RyR2 resulting in further calcium‐induced calcium release, and gain‐of‐function of L‐type calcium channels and sarcoplasmic endoplasmic reticulum calcium channel (SERCA).^32^ The net effect of these pathways results in Ca^2+^ overload within the cardiomyocyte, causing an increased propensity toward developing EAD and DAD, both of which are prerequisites for developing arrhythmias such as premature ventricular complex (PVC), premature atrial complex (PAC), and even more life‐threatening arrhythmias like VT or VF.^32^, ^33^ In addition, the use of pharmacological treatments for COVID‐19, such as antimalarial agents (hydroxychloroquine/chloroquine) and anti‐viral agents (lopinavir/ritonavir), has been shown to further prolong the QTc interval through inhibition of the hERG‐potassium channel and inhibition of the enzyme cytochrome 450, thereby increasing the risk of QT‐related life‐threatening ventricular arrhythmias, particularly TdP.^34^ Macrolides such as azithromycin and clarithromycin, which are frequently administered to prevent lung bacterial superinfection, have also been reported to prolong the QT interval and increase the risk of TdP.^34^, ^35^ Given the wide variety of pharmaceutical and medical approaches in treating COVID‐19 infection, pharmacokinetic and pharmacodynamic drug interactions are needed to be considered to minimize the risk of cardiac arrhythmias.

COVID‐19 patients experienced increased heart rate as the most common finding of rhythm disturbances on hospital admission.^36^, ^37^ The increased heart rate also the most common ECG abnormalities in the patient with SARS, with the incidence of around 72%.^38^ The present meta‐analysis showed that COVID‐19 patients with increased heart rate tend to have a poor outcome. Consistent with this finding, a previous study showed that COVID‐19 patients who need to be treated in the ICU have a faster heart rate compared with the general ward.^37^ A study related to COVID‐19 mortality also showed that non‐survivor have significantly faster baseline heart rates on admission compared with survivors.^39^ The increased heart rate might be related to the increased risk of atrial tachyarrhythmias, which were common in COVID‐19 patients admitted to the ICU and often followed by hemodynamic deterioration, thus leading to poor outcomes.^4^ The mechanisms that underlie atrial tachyarrhythmias and tachycardia in these patients may be due to systemic infection, direct viral cardiomyocyte injury, hypoxia, and natural susceptibility of aged, comorbid‐laden individuals.^40^ Hypoxia has been shown to directly cause tachycardia in human studies involving spectral analysis of R‐to‐R interval series. Hypoxia was shown to attenuate autonomic nervous system activities with the sympathovagal balance leaning more heavily toward sympathetic dominance.^41^

The present meta‐analysis showed that COVID‐19 patients with longer QRS duration and incidence of LBBB tend to have poor outcomes. In COVID‐19 patients, longer QRS duration and the presence of LBBB may indicate intraventricular conduction delay, which can be a sign of myocardial injury and led to pump failure, which is independently associated with death.^14^, ^17^ Similarly, patients with myocarditis with a prolonged QRS complex was associated with lower left ventricular function and higher cardiovascular mortality.^42^

The present study also showed that the presence of PAC and PVC on admission ECG was more frequent in COVID‐19 patients with poor outcomes. As previously explained, infection of SARS‐COV2 triggers overexpression of AngII, which subsequently causes dysfunctional CAMCK‐II activity downstream and eventually PAC and PVCs.^25^, ^26^, ^27^, ^28^, ^29^, ^30^ Besides this, the appearance of PAC may also be caused by transient systolic and diastolic dysfunction due to cytokine hypersecretion in COVID‐19 patients.^43^ The presence of a PAC detected on baseline ECG recording was associated with an increased risk of developing AF, which could increase the risk of congestive heart failure, ischemic heart disease, and sudden cardiac death.^43^, ^44^ Aside from that, the presence of PVC has been detected in 4.4% up to 5% of COVID‐19 patients undergoing standard 12‐leads ECG on admission.^13^, ^15^ The inflammatory process in COVID‐19 is also considered to play a role in the incidence of PVC. A retrospective study of 264 patients undergoing ambulatory Holter ECG monitoring showed that the neutrophil‐lymphocyte ratio (NLR) was found higher in the PVC group and was independently associated with the presence of PVC, suggesting the role of the inflammatory cytokine storm.^45^ The PVC existence may also represent an underlying disease that indirectly explains the role of PVC in increasing poor outcomes in COVID‐19 patients through the involvement of heart failure. A cohort study conducted by Atherosclerosis Risk in Communities (ARIC) shows that PVC is associated with the prevalence of heart failure.^46^ Other than these mechanisms, PVC will eventually increase the risk of more malignant dysrhythmias such as sustained VT or VF, which leads to sudden cardiac death.^47^

Another ECG manifestation of cardiac involvement in COVID‐19 with poor outcome in the present study is ST‐segment/T‐wave abnormalities. Generally, ST‐segment depression and T‐wave inversion represent myocardial ischemia, whereas ST‐segment elevation represents an ongoing myocardial injury.^46^ COVID‐19 patients reveal that mononuclear cells infiltration in the myocardium, suggesting the role of cytokine storm toward myocarditis in COVID‐19 infection. T‐wave inversion might be an early warning of myocarditis, as the appearance of T‐wave inversion has been associated with myocardial edema on cardiac MRI of myocarditis patients.^47^ Meanwhile, ST‐segment depression detected on the ECG is both markers of cardiac injury and poor prognosis for COVID‐19 patients.^48^ A cohort study of COVID‐19 patients with a follow‐up up to 45 days shows that T‐wave inversion (≥1 mm) and ST‐depression (≥0.5 mm) as independent predictors of death.^49^ Interestingly, several studies have shown a link between severe COVID‐19 infection with electrolyte imbalance, namely hypokalemia, and hypomagnesemia, possibly mediated through gastrointestinal and renal loss.^50^, ^51^ Both of these electrolyte imbalances have been shown to attenuate cardiomyocyte depolarization and result in QTc prolongation and ST waveform changes, as seen in the poor outcome arm of this cohort.^52^, ^53^

## 5 LIMITATION

There are several limitations to this study. *First*, all included studies had a retrospective study design, and the data were not sufficiently matched or adjusted for confounders. Therefore, the ECG parameters may be affected by differences in patients’ severity at admission. *Second*, there are some variations of cut‐off points for prolonged QTc intervals in different studies and the limitation of Bazett's formula in correcting the QT‐interval. Bazett's formula may lead to overcorrecting the QTc value when used at high heart rates.^54^ Since both higher heart rates and prolonged QTc intervals are significantly associated with increased poor outcomes in COVID‐19 patients, the effect of prolonged QTc intervals in poor outcomes may be exaggerated by Bazett's formula overcorrecting the QT interval.

## 6 CONCLUSION

This meta‐analysis showed ECG abnormalities on admission, including longer QTc interval and prolonged QTc interval, longer QRS duration, a faster heart rate, the presence of LBBB, PAC, PVC, T‐wave inversion, and ST‐depression are significantly associated with an increased composite poor outcome in patients with COVID‐19.

## 7 CLINICAL IMPLICATION

- Several ECG abnormalities on admission (longer QTc interval, prolonged QTc interval, longer QRS duration, faster heart rate, LBBB, PAC, PVC, T‐wave inversion, and ST‐depression) are associated with poor outcome in COVID‐19 patients.
- Risk stratification of COVID‐19 patients must be done early, and admission ECG can be used to identify the underlying disease.
- In patients with prolonged QTc intervals at the baseline and patients with inherited arrhythmic syndromes, ECG should be evaluated and monitored regularly.

## CONFLICTING OF INTERESTS

The authors declare no conflict of interest for this article.

## ETHICS APPROVAL

Not applicable.

## Supporting information

Supplementary Material
