# Supplementary material for: Electrocardiography on admission is associated with poor outcomes in coronavirus disease 2019 (COVID‐19) patients: A systematic review and meta‐analysis
Source: J Arrhythm. 2021 Jun 14;37(4):877–85. doi: 10.1002/joa3.12573 (PMC8339106; doi:10.1002/joa3.12573)
Supplement: Supplementary file 1 — Supplementary Material [file JOA3-37-877-s001.pdf]

Supplementary data

Table S1. Details of the search strategy.

No.	Database	Search strategy
1	MEDLINE	1. (COVID-19) OR (coronavirus disease 2019) OR (COVID 19) OR COVID19 OR "2019 novel coronavirus" OR 2019nCoV OR (2019 nCoV) OR "new coronavirus" OR (Wuhan AND coronavirus) OR (SARS CoV-2) OR "novel coronavirus" OR (SARS-CoV-2) OR (2019-nCoV)<br>2. (Electrocardiogra* OR ECG OR EKG).ti. ab .<br>3. Prognos* OR (prognostic factor*) OR mortal* OR death OR sever* OR (critically ill)<br>4. #1 and #2 and #3 and 2020/01/01:2020/11/01[dp]
2	Cochrane Central Database	(2019 nCoV) OR 2019nCoV OR "2019 novel coronavirus" OR (COVID-19) OR COVID19 OR "new coronavirus" OR "novel coronavirus" OR (SARS CoV-2) OR (Wuhan AND coronavirus) OR (COVID 19) OR (2019 nCoV) OR (SARS-CoV-2) OR (coronavirus disease 2019) in All Text AND Electrocardiogra* OR ECG OR EKG in Title Abstract Keyword AND Prognos* OR (prognostic factor*) OR mortal* OR death OR sever* OR (critically ill) in All Text - with Cochrane Library publication date Between Jan 2020 and Nov 2020
3	Europe PMC	((COVID-19) AND (electrocardiogram or electrocardiography or electrocardiographic) AND (mortality OR death OR severe OR severity OR critically ill) AND (FIRST_PDATE:[2020-01-01 TO 2020-11-01])

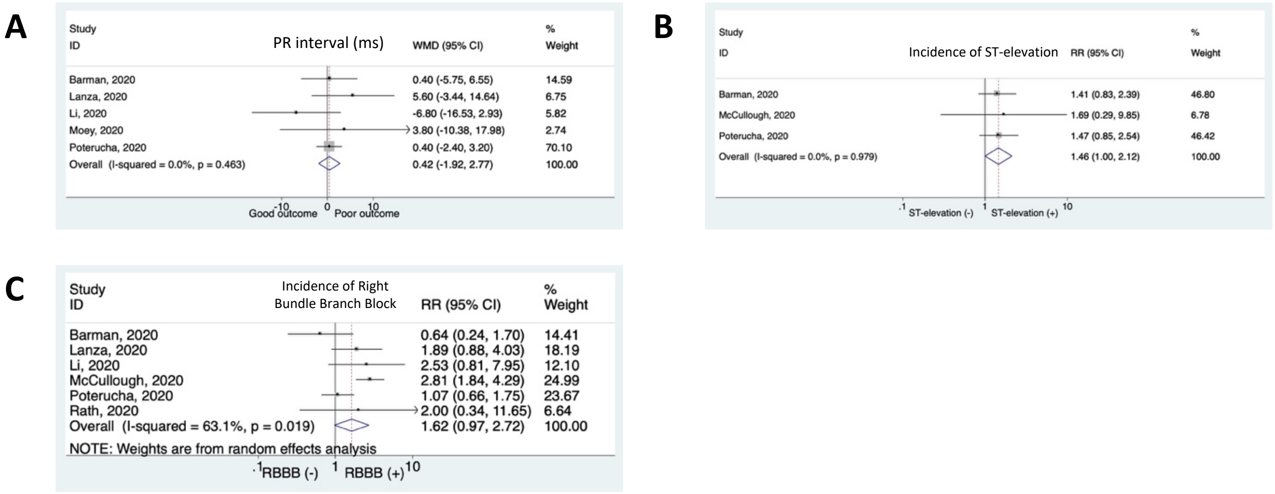

Figure S1. Several ECG findings and the outcome of COVID-19. (A) a longer PR interval, (B) right bundle branch block, and (C) ST-elevation are not associated with increased risk of composite poor outcome.

Table S2. Assessment of included studies in the Newcastle-Ottawa Scale (NOS).

No	Study	Selection (max 4 stars)	Comparability (max 2 stars)	Outcome (max 3 stars)
1	Barman, 2020	****	**	***
2	Lanza 2020	****	**	***
3	Li, 2020	****	**	***
4	McCullough, 2020	****	**	***
5	Moey, 2020	****	*	***
6	Poterucha, 2020	****	**	**
7	Rath, 2020	****	**	**
